# Selective Enrichment of Antibacterial Peptides from Chicken Hemoglobin Hydrolysates by Electrodialysis with Ultrafiltration Membranes (EDUF)

**DOI:** 10.3390/molecules31071184

**Published:** 2026-04-02

**Authors:** Delasa Rahimi, Sergey Mikhaylin, Laurent Bazinet

**Affiliations:** 1 Institute of Nutrition and Functional Foods (INAF), Université Laval, Quebec City, QC G1V 0A6, Canada; delasa.rahimi-galougahi.1@ulaval.ca (D.R.); sergey.mikhaylin@fsaa.ulaval.ca (S.M.); 2 Food Science Department, Université Laval, Quebec City, QC G1V 0A6, Canada; 3 Laboratoire de Transformation Alimentaire et Procédés ÉlectroMembranaires (LTAPEM, Laboratory of Food Processing and Electro-Membrane Processes), Food Science Department, Université Laval, Quebec City, QC G1V 0A6, Canada; 4 PROTEO, The Quebec Network for Research on Protein Function, Engineering, and Applications, Montréal, QC H2X 3Y7, Canada; 5 PROTEO-ULaval, Centre de Recherche sur les Protéines, Université Laval, Quebec City, QC G1V 0A6, Canada; 6 Laboratory of Food Sustainability (EcoFoodLab), Food Science Department, Université Laval, Quebec City, QC G1V 0A6, Canada

**Keywords:** bioactive peptides, antifungal activity, chicken hemoglobin, electrodialysis with ultrafiltration membranes, fractionation, circular bioeconomy

## Abstract

Chicken hemoglobin represents a source of bioactive peptides that could replace synthetic additives. This study evaluated the antibacterial and antifungal potential of chicken hemoglobin hydrolysates and the effect of their fractionation by EDUF. Hemoglobin was hydrolyzed with pepsin at pH 3 for 0.5 h and 6 h, followed by discoloration, and then fractionated by EDUF for 180 min at pH 7. Fractions were characterized using RP-UPLC-MS/MS, and antimicrobial activity was assessed. Antibacterial activity against *Escherichia coli* was observed only in EDUF fractions (P+180 and P−180), while crude hydrolysates showed no effect. However, MIC values of these EDUF fractions indicated weak inhibition. Antifungal activity was primarily detected in the final feed fractions against *Mucor racemosus* and *Rhodotorula mucilaginosa* (MIC: 0.04–20.00 mg/mL). Database matching of the fractions identified 22 sequences corresponding to peptides previously reported as bioactive, including ALARL, FDK, LARL, and VVYPW, which have been associated with antioxidant, ACE-inhibitory, antihypertensive, and enzyme-inhibitory properties. Nevertheless, EDUF proved to be an efficient, solvent-free, and low-energy approach for the recovery of peptide fractions from chicken hemoglobin, supporting the potential development of natural bioactive ingredients within a circular bioeconomy. Modifications of EDUF parameters, such as membrane configuration, pH, and voltage, could further enhance peptide selective recovery and the enrichment of functional fractions.

## 1. Introduction

Proteins are not only key nutrients but also sources of bioactive peptides (BPs), small fragments released during protein digestion or hydrolysis. These peptides, present in animal blood and characterized by low molecular masses, exhibit diverse properties such as antimicrobial [1], antioxidant [2], antihypertensive [3], and antidiabetic effects [4]. In recent years, increasing attention has been directed toward natural bioactive compounds due to growing concerns regarding the use of chemical preservatives, which have been associated with adverse health effects, including allergies, hyperactivity, asthma, headaches, and vomiting, as well as potential mutagenic and carcinogenic risks such as leukemia and colon cancer [5,6]. In contrast, BPs have gained increasing interest because of their multifunctional properties, safety, and potential applications as natural ingredients in functional foods and nutraceuticals [7,8]. Among the wide range of bioactivities described for these peptides, their antimicrobial potential is of particular relevance [9]. Antimicrobial peptides (AMPs), also known as host defense peptides, are naturally occurring molecules that form part of the innate immune system in many organisms. They exhibit broad-spectrum activity against bacteria, fungi, and viruses through diverse mechanisms, such as disrupting microbial membranes or targeting intracellular processes, which reduces the likelihood of resistance development [10,11]. This property is especially important in the context of the rising threat of multidrug-resistant pathogens and the limited pipeline of novel antibiotics. Consequently, identifying new AMPs from unconventional protein sources has become a promising strategy for both therapeutic and food preservation applications [12].

While many dietary protein sources have been studied, the exploration of by-products as potential sources of BPs remains less developed. Slaughterhouse by-products, especially blood-derived fractions, are increasingly recognized as valuable substrates for generating bioactive compounds, supporting circular economy initiatives in the food sector [13,14]. Among these, the red cell-rich fraction of blood, primarily composed of hemoglobin, represents a promising nutritional and functional source owing to its high protein content and underutilization in current processing chains. In Canada alone, nearly 48 million liters of chicken blood are produced each year, yet its transformation into AMPs remains significantly underexplored [1]. Prior research has illustrated the potential of bovine and porcine hemoglobin as sources of antimicrobial and antioxidant peptides. For instance, hemoglobin-derived peptides such as α137–141 (Neokyotorphin) have been identified in bovine hemoglobin. These peptides exhibit broad-spectrum antimicrobial activity and antioxidative effects, making them appropriate for food preservation applications [15,16]. Similarly, a recent work on porcine hemoglobin has identified novel antifungal peptides. García-Vela et al. synthesized sixteen peptides, among which five peptides, including the alpha-helical FQKVVAGVANALAHKYH, demonstrated broad antifungal activity against common spoilage fungi such as *R. mucilaginosa* and *Paecilomyces* spp. [17]. Studies on chicken hemoglobin are more recent but are rapidly advancing. Using machine-learning-guided peptide identification, Rahimi et al. showed that 30 min chicken hemoglobin hydrolysates displayed strong antibacterial and antifungal activities, with inhibition zones up to 19.10 mm. Hydrolysis at acidic pH (2–3) produced higher degrees of hydrolysis and effective antifungal activity. Partial Least Squares Discriminant Analysis (PLS-DA) identified 31 peptide candidates, including LARKYH and DLSHGSAQIKGHGKKVVAAL as antifungal peptides. However, no antibacterial peptides were detected among the synthesized peptides [1].

Although previous studies have identified antibacterial peptides from chicken blood, efficiently separating and enriching these active peptides from complex hydrolysate mixtures remains a challenge. To enhance the bioactivity of such complex hydrolysates, purification approaches can be applied to remove compounds that may exert antagonistic or anti-synergistic effects on the peptides. Various methods have been used to separate or purify these complex hydrolysates in order to improve their bioactivity, including reverse-phase high-performance liquid chromatography (RP-HPLC) [18,19], which provides high resolution but is costly and unsuitable for large-scale use, and ultrafiltration pressure-driven processes [20], which are less expensive but limited in selectivity since they separate peptides only by molecular mass. Eco-efficient approaches such as electrodialysis and its associated technologies have been recognized as green technologies capable of separating ionic compounds by applying an electric field across ion-exchange membranes [21,22]. However, most conventional methods lack the necessary selectivity to efficiently separate peptides with similar sizes but different charges, or vice versa, often requiring multiple processes [23].

In this context, EDUF represents a promising separation technology for the fractionation of bioactive compounds. EDUF combines the principles of conventional electrodialysis and membrane filtration by integrating ultrafiltration membranes with ion-exchange membranes in an electrically driven system [24]. Under an applied electric field, charged molecules such as peptides migrate through filtration membranes according to their charge, while the ultrafiltration membranes simultaneously provide size-based selectivity by restricting the passage of larger molecules. This dual separation mechanism enables the selective migration and fractionation of peptides according to both their electrical charge and molecular weight. By adjusting operational parameters such as the electric field strength, pH, and membrane characteristics, EDUF can be optimized to selectively recover and enrich target peptides. Owing to these advantages, EDUF has been successfully applied to the separation of bioactive peptides [25] and to enrich hemoglobin-derived antimicrobial peptide from bovine hydrolysates (α137–141) [16]. Furthermore, the antihypertensive activity of the EDUF fractions derived from chicken by-products (chicken heart and liver) was studied by Adaile-Pérez et al. [26]. However, the application of EDUF to chicken hemoglobin-derived peptides has not yet been explored. To date, no studies have reported the use of EDUF for the fractionation of chicken hemoglobin. Consequently, the peptide profiles and the antimicrobial potential of the resulting fractions remain largely unexplored. Notably, this study represents the first application of EDUF for the fractionation of chicken hemoglobin hydrolysates. In addition, it provides the first comparison of two peptic hydrolysis durations (0.5 h and 6 h) within this separation workflow in order to evaluate how hydrolysis time influences peptide separation by EDUF and consequently their peptide mass profile distributions and antimicrobial activity. The objectives of this work were therefore: (1) to evaluate the feasibility of EDUF for the separation of peptides from chicken hemoglobin hydrolysates and to examine its potential influence on antibacterial and antifungal activities; (2) to assess the effect of hydrolysis duration (0.5 h and 6 h) on the properties and peptide populations of the resulting fractions; and (3) to identify candidate peptides associated with additional reported bioactivities, such as antioxidant and antihypertensive activities, in the fractions obtained after EDUF. By investigating the valorization of chicken hemoglobin and a solvent-free, low-energy separation approach, this study provides foundational information that may support future research on natural, clean-label ingredients.

## 2. Results and Discussion

### 2.1. Degree of Hydrolysis

**Figure 1:**
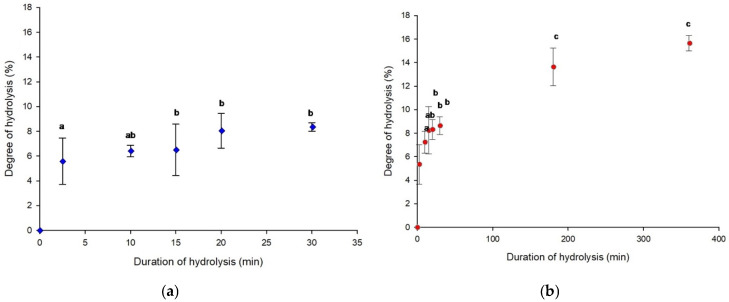
Degree of hydrolysis during peptic hydrolysis of chicken hemoglobin at pH 3 after 0.5 h (**a**) and 6 h (**b**). Error bars represent standard deviation. Values with different letters are significantly different according to Tukey’s test (*p* < 0.05).

As shown in Figure 1a, b, the degree of hydrolysis (DH) increased over the duration of hydrolysis at both 0.5 h and 6 h, respectively. For the 0.5 h hydrolysis, DH reached 8.35 ± 0.35% after 30 min, with no significant differences compared with the DH values at 15 and 20 min (*p* > 0.05). Also, the 6 h hydrolysis allowed to reach a DH% of 15.65 ± 0.66, which was not significantly different from the value obtained at 3 h (13.64 ± 1.61) (*p* > 0.05).

These results show that DH increased during hydrolysis, indicating that larger peptides were progressively broken down into smaller peptides or free amino acids. This behavior is consistent with the classical “zipper” mechanism of pepsin, in which the enzyme progressively cleaves peptide bonds along unfolded protein chains, resulting in the gradual formation of smaller peptides [27]. In previous studies, the DH of chicken hemoglobin hydrolysates at pH 3 after 3 h was reported at 11.44 ± 1.49% [1], which is slightly lower than the present value (13.64 ± 1.61%). Rahimi et al. found 10.44 ± 1.0% after 0.5 h and 18.61 ± 0.66% after 6 h, the latter being somewhat higher than the result of the current study (15.65 ± 0.66%) [28]. This difference is probably related to the larger hydrolysis volume and the use of a magnetic stirrer instead of a shaking incubator. Zouari & Deracinois also reported a DH% of 11.8% for chicken hemoglobin after 24 h of hydrolysis at pH 3 and 23 °C [29]. Hydrolysis duration is a key factor influencing the DH%, peptide population generated and, consequently, the bioactivity of protein hydrolysates. Different hydrolysis times can lead to the release of distinct peptides or further degradation of previously generated ones, thereby modifying biological activity [30]. For example, whey protein hydrolysates produced under optimized conditions exhibited ACE inhibitory IC_50_ values ranging from 0.202 mg mL^−1^ after 1 h to 0.148 mg mL^−1^ after 5 h [31]. Similarly, significant differences in Neprilysin inhibitory activity have been observed among hydrolysates obtained at different hydrolysis times (2, 6, and 24 h), highlighting the strong influence of hydrolysis duration on the resulting bioactive peptide profile [32]. Overall, these comparisons confirm that the present results are consistent with previous findings and highlight the strong influence of hydrolysis duration, temperature, and other minor experimental changes on the degree of hydrolysis.

### 2.2. Protein Content and Number of Peptides

**Figure 2:**
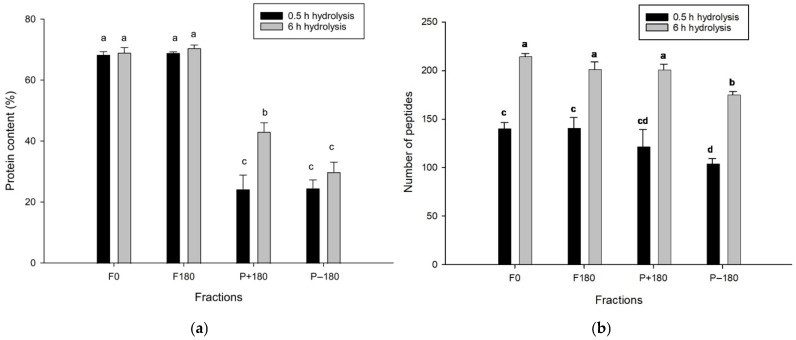
(**a**) Protein content (%) determined by the Dumas method and (**b**) number of peptides identified by RP-UPLC-MS/MS in fractions before and after EDUF for 0.5 h and 6 h discolored hydrolysates. Different letters indicate statistical differences between values in different fractions before and after EDUF according to Tukey’s test (*p* < 0.05).

The protein content and the number of peptides in the initial feeds (F0-0.5 h and F0-6 h) and in the three EDUF compartments—the final feed (F180-0.5 h and F180-6 h), the positively charged peptide recovery compartment (P+180-0.5 h and P+180-6 h), and the negatively charged peptide recovery compartment (P−180-0.5 h and P−180-6 h)—are presented in Figure 2a,b. Overall, all fractions showed a significant increase in the number of peptides from 0.5 h to 6 h, confirming the strong influence of hydrolysis duration on peptide generation and reflecting the progressive breakdown of larger peptides into smaller ones [33].

Regarding protein content, the P+180 and P−180 fractions exhibited significantly lower values than both the initial and final feeds at each hydrolysis duration. This result indicates that only a portion of the peptides migrated into the cationic and anionic compartments during EDUF, while most peptides remained in the feed. For the 0.5 h hydrolysis, F0 and F180 showed similar protein levels, both significantly higher than those measured in P+180 and P−180, which did not differ from each other. After 6 h of hydrolysis, the protein content increased significantly in the P+180 fraction (43.09 ± 2.93%), whereas the P−180 fraction remained lower (29.89 ± 3.14%), suggesting greater peptide migration toward the cationic compartment as hydrolysis progressed.

Although the lower protein content was observed in the P+180 fraction, the total number of peptides remained statistically similar to that observed in the feed compartments. This apparent discrepancy can be attributed to the compositional complexity of the feed, which contains peptides of various sizes, free amino acids, and very small peptides (<3 amino acids). These small compounds contribute to nitrogen measurements, thereby increasing the apparent protein content, but are not counted in peptide-number analyses, as they fall below the identification threshold of ≥3 amino acid residues.

For both 0.5 h and 6 h hydrolysis durations, the P−180 fractions contained significantly fewer peptides than F0 and F180, indicating selective migration of negatively charged peptides into the anionic compartment.

### 2.3. Peptides Mass Distribution

**Figure 3:**
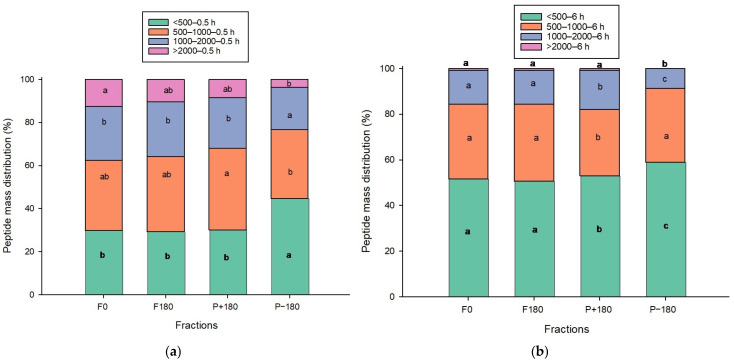
(**a**) Peptide mass distribution before and after EDUF in different recovery compartments for 0.5 h, (**b**) and 6 h discolored hydrolysates. Different letters indicate statistical differences between values with the same molecular mass in different EDUF fractions for 0.5 h and 6 h according to Tukey’s test (*p* < 0.05).

The peptide mass distribution in the different compartments before and after EDUF for the 0.5 h and 6 h discolored hydrolysates is shown in Figure 3a, b. For the 0.5 h discolored hydrolysate, no significant changes were observed for peptides >2000 Da in the feed (*p* > 0.05). Peptides with higher molecular masses (1000–2000 Da) migrated less toward the P−180 compartment after EDUF, while peptides in the 500–1000 Da range migrated more toward P+180 than P−180. In contrast, peptides <500 Da predominantly migrated into the P−180 fraction, and these differences were significant (*p* < 0.05). This pattern suggests that part of the larger peptides migrated preferentially toward the P+180 compartment rather than the P−180 compartment.

For the 6 h discolored hydrolysate (Figure 3b), peptides >2000 Da represented less than 1% of the initial feed. The peptide mass distribution across all molecular mass ranges in F0 and F180 did not differ significantly (*p* > 0.05). However, the proportion of peptides <500 Da was significantly higher in the final anionic compartment (P−180) than in the other fractions (*p* < 0.05). In contrast, the percentage of peptides in the 1000–2000 Da range was significantly lower in P−180. Conversely, more peptides in the 1000–2000 Da and 500–1000 Da ranges migrated into P+180. These differences were statistically significant (*p* < 0.05).

These results suggest that larger peptides (>2000 and 1000–2000 Da) may carry more positive charges, facilitating their migration toward the P+180 compartment during the 180 min of EDUF, particularly in the 6 h discolored hydrolysate. Previous studies have shown that higher-molecular-mass peptides are progressively broken down into smaller fragments during hydrolysis at pH 3, consistent with the zipper mechanism of pepsin [33], allowing smaller and highly charged peptides to migrate more easily through the membrane [34].

### 2.4. Membrane Integrity

Changes in the physicochemical properties of the membranes, particularly their thickness and electrical conductivity, were evaluated to assess potential fouling during the fractionation process. Monitoring these parameters is important because fouling can alter ion transport properties and reduce separation efficiency in membrane-based systems [35]. 

**Figure 4:**
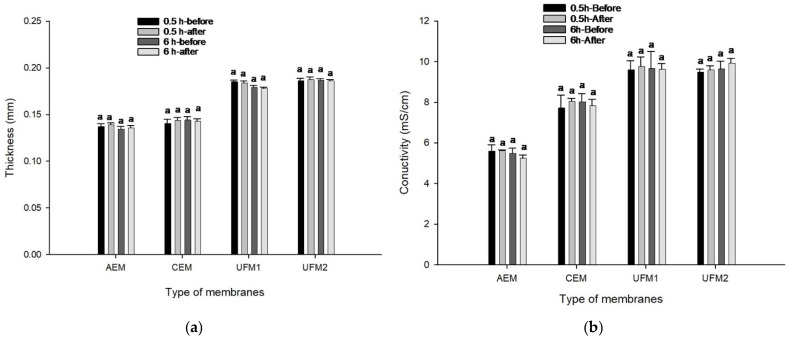
Membrane characterization before and after EDUF treatment: (**a**) thickness (mm), and (**b**) conductivity (mS/cm) for 0.5 h and 6 h discolored hydrolysates. Similar letters indicate no statistical differences between the values measured before and after EDUF treatment for each membrane under both conditions, according to Tukey’s test (*p* > 0.05).

Figure 4a shows that the thickness of the cationic, anionic, and filtration membranes did not change after EDUF and was not significantly different for either the 0.5 h or 6 h discolored hydrolysates (*p* > 0.05). Similarly, membrane conductivity (Figure 4b) remained unchanged before and after EDUF for both hydrolysis durations. These results suggest that no detectable fouling occurred on the membranes, regardless of the differences in the size and distribution of the peptides, and that peptides from the discolored hydrolysates migrated efficiently through the different membranes. Consistent with previous studies on bovine hemoglobin using EDUF [27] and the findings of Przybylski et al. [36] no significant changes in membrane thickness or electrical conductivity were observed before and after EDUF, confirming the absence of membrane fouling for the discolored chicken hemoglobin hydrolysates. However, prior to discoloration, fractionation of the hemoglobin hydrolysates was not feasible, as the presence of haem–peptides or peptide–peptide aggregates caused pronounced membrane fouling, as demonstrated by Vanhoute et al. on bovine hemoglobin [27]. Therefore, the absence of significant changes in these properties suggests that the membranes did not experience detectable fouling under the experimental conditions used in this study. This observation suggests that the EDUF process remained stable during the experimental period. However, further studies conducted over longer operating periods and under industrial conditions would be required to confirm membrane stability and to evaluate potential implications for cleaning frequency and membrane replacement [37].

### 2.5. Peptide Concentration and Migration Rate

**Figure 5:**
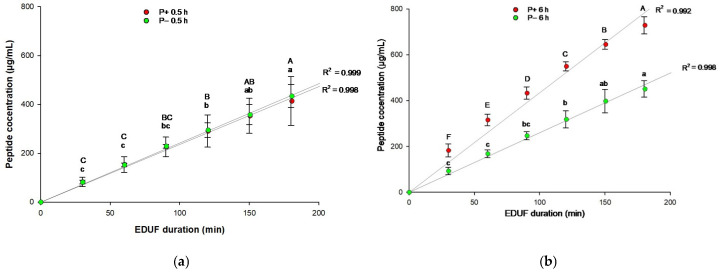
Peptide concentration in P+180 and P−180 for (**a**) 0.5 h and (**b**) 6 h discolored hydrolysates. Values with different uppercase and lowercase letters within each compartment (P+ and P−, respectively) at the same duration point indicate significant differences (Tukey’s test, *p* < 0.05).

As shown in Figure 5, peptide concentration increased throughout the 180 min EDUF process. For the 0.5 h discolored hydrolysate, no significant difference was observed between the peptide concentrations of the P+180 and P−180 compartments (*p* > 0.05). However, for the 6 h discolored hydrolysate, a significant difference was found between P−180 and P+180 (*p* < 0.05), with more peptides migrating to the P+180 than to the P−180. This result is consistent with previous results on protein content (Figure 5a). This observation can be explained by the greater abundance of smaller peptides generated after 6 h of hydrolysis, which results from the progressive cleavage of higher-molecular-mass peptides. According to Cournoyer et al., the strong influence of molecular weight on peptide migration indicates that low-MW peptides undergo rapid charge reorientation under an alternating electric field, enabling them to migrate more readily than larger peptides [38]. Consistent with this phenomenon, extending the hydrolysis duration significantly increased (*p* < 0.05) the migration rate toward the cationic compartment (P+180), reaching 17.67 ± 4.70 g/m^2^·h after 6 h compared with 8.68 ± 1.48 g/m^2^·h after 0.5 h. In contrast, the migration rate toward the anionic compartment (P−180) did not differ significantly (*p* > 0.05) between 0.5 h (8.28 ± 0.85 g/m^2^·h) and 6 h (9.22 ± 0.86 g/m^2^·h). These findings indicate that prolonged hydrolysis promotes the migration of smaller, positively charged peptides, while the migration of negatively charged peptides remains largely unaffected, regardless of their molecular size.

### 2.6. Hierarchical Clustering Analysis

**Figure 6:**
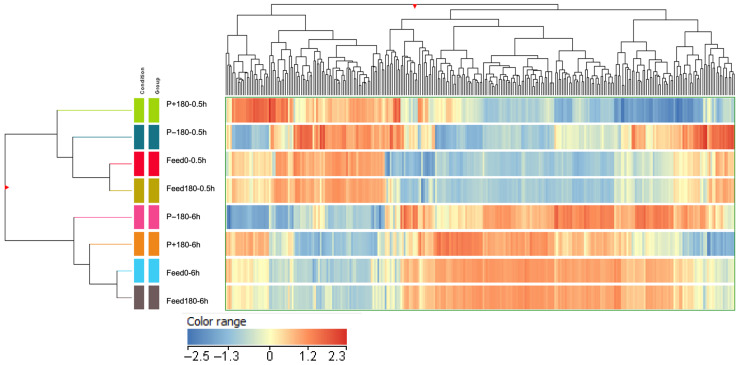
Hierarchical clustering of EDUF fractions (F0, F180, P+180, and P−180) for 0.5 h and 6 h discolored hydrolysates, with similarities measured by Euclidean distance and peptide profiles displayed as a heat map. The color scale ranges from cooler tones (blue) to warmer tones (red), representing low to high peptide intensities, respectively, with color intensity reflecting the degree of upregulation or downregulation.

Figure 6 shows the hierarchical clustering analysis (HCA) performed to evaluate the effect of EDUF fractionation on peptide profiles compared to the initial feed for 0.5 h and 6 h discolored hydrolysates, as first applied by Cournoyer, Daigle et al. [24]. On the left side of Figure 6, seven distinct clusters represent the similarities in peptide composition among fractions. The main cluster clearly separates the peptide populations of the 0.5 h and 6 h discolored hydrolysates, underscoring the significant impact of hydrolysis duration on peptide composition, as further evidenced by the largest Euclidean distance observed between their initial feeds. The closest Euclidean distance was observed between the initial and final feed fractions for both 0.5 h and 6 h discolored hydrolysates, indicating the lowest changes in peptide composition after EDUF in the feed. This was followed by P−180 for the 0.5 h discolored hydrolysate and P+180 for the 6 h discolored hydrolysate.

The lowest similarity among the 0.5 h fractions was observed in P+180, suggesting the greatest changes compared to the initial feed, as peptides migrated more extensively and concentrated in this compartment. In contrast, for the 6 h discolored hydrolysate, the P−180 fraction showed the greatest dissimilarity. This can be explained by the effect of hydrolysis duration on peptide composition. A longer hydrolysis duration produces smaller peptides (Figure 3b) with different charge distributions, which in turn alter their migration behavior during EDUF. As a result, positively charged peptides become relatively more abundant after extended hydrolysis and preferentially migrate toward the cationic side (P+180), making this fraction more similar to the feed. This also explains why the P−180 fraction is the most dissimilar compared to the others. Table 1 presents the abundance of peptides with potential antimicrobial activity under different conditions. Most of these peptides were more abundant in the P+0.5 h and P+6 h fractions, likely because AMPs are typically identified in databases based on their positive net charge. Consequently, positively charged peptides were predominantly detected in the cationic compartment fractions. Although their molecular masses varied, this parameter did not appear to have a strong influence on migration in this study, as peptides of different molecular masses were detected across all fractions. This can be attributed to the large pore size (50 kDa) of the membranes, which facilitates the migration of small peptides (≤2 kDa). Nevertheless, previous studies have reported that the membrane molecular weight cut-off (MWCO) can influence peptide selectivity depending on their size, charge and conformation, suggesting that under different operating conditions, peptide MW and resulting conformation could still play a role in peptide fractionation [38,39,40].

In addition, several peptides showing sequence similarity to known AMPs were identified across different fractions. Among them, DLSHGSAQIKGHGKKVVA and DLSHGSAQIKGHGKKVVAAL (with matching scores of 0.9 and 1.0 with DLSHGSAQIKGHGKKVVAAL, respectively, reported by Rahimi et al. [1]), as well as HAHKLRVDPVN and HKLRVDPVN (with matching scores of 0.9 and 1.0 with HAHKLRVDPVN, (Rahimi et al. [28]), were found exclusively in the P−180 fractions, particularly after 0.5 h and 6 h of hydrolysis. These longer peptides (net charge +1.0 to +2.2) likely migrated toward the anionic membrane due to electrostatic or hydrophobic interactions with other peptides present. Indeed, previous studies have shown that neutral or cationic peptides can migrate toward the anionic compartment by forming aggregates with oppositely charged peptides through hydrophobic and electrostatic interactions. Such interactions can lead to globally positive or neutral peptide complexes that behave differently under an electric field, allowing partial migration toward the anionic compartment. Therefore, the presence of these positively charged peptides in the P−180 fraction may result from peptide–peptide aggregation or “salting-out” effects that promote their transport as larger complexes rather than individual molecules [41]. In contrast, the P+180 fractions were enriched with a greater variety of shorter, positively charged peptides such as ALARL, HALARKY, HGKKVL, VRVVAHALARKYH, and VRGHGKKVLGAL (like some antimicrobial peptides identified from turkey hemoglobin, unpublished data from our team). These peptides, with molecular masses below 1 kDa and higher net positive charges, migrated efficiently toward the cationic compartment. This behavior is consistent with the main physicochemical factors governing peptide migration during EDUF. Peptides charge, molecular mass, hydrophobicity, and isoelectric point collectively influence their electrophoretic mobility and selective transfer through ultrafiltration membranes [38]. A recent work using regression-tree modeling confirmed that peptide isoelectric point and molecular mass are the most influential variables in predicting migration rate, followed by hydrophobicity (GRAVY score) [38]. Peptides with higher pI values (>9.7) and smaller sizes (4–17 amino acids, 550–1850 Da) exhibited the highest migration efficiencies, particularly those containing antimicrobial amino acid sequences such as TSKYR and STVLTSKYR. As illustrated by their regression tree under continuous current, even though the model did not split directly on GRAVY score, most low-molecular-mass peptides (<738 Da) with pI values between 5.3 and 8.3 showed positive GRAVY scores, indicating hydrophobic character. These results highlight that hydrophobic interactions occurred between peptides, helping explain the higher migration rate of such species.

Overall, their continuous current regression tree model revealed that the peptide pI, defining its charge relative to the process pH, had the strongest influence on migration, followed by molecular mass, consistent with the EDUF principle of separating molecules according to their charge (electric-field driving force) and size (membrane cut-off). Positively charged peptides migrated the most, while hydrophobic and electrostatic peptide–peptide interactions further facilitated the migration of slightly negative or neutral peptides by forming globally positive aggregates. In the present work, the EDUF process effectively concentrates AMP candidates according to their physicochemical features and hydrolysis duration: most of the short, cationic peptides accumulate in P+180, whereas longer and positively charged ones migrate toward P−180 or remain in the feed.

### 2.7. Antimicrobial Activity

#### 2.7.1. Diffusion Test

Table 2 presents the inhibition zone diameters of hydrolysates and EDUF fractions against fungal and bacterial strains. Among the fungi, *M. racemosus* showed the lowest sensitivity to all treatments, including the crude hydrolysates, whereas *Paecilomyces* spp. and *R. mucilaginosa* were more susceptible. The inhibition zone against *Paecilomyces* spp. ranged from 9.92 ± 0.61 mm (F0-6 h) to 14.47 ± 0.19 mm (F0-0.5 h), confirming that shorter hydrolysis durations generating longer peptides provide hydrolysates with stronger antifungal activity [1,42].

After EDUF, the F180-0.5 h fraction maintained a similar inhibition zone to its initial feed, while F180-6 h displayed slightly higher activity than F0-6 h, indicating that EDUF can selectively enrich bioactive peptides. Among the separated compartments, only the P+180 fractions, enriched in cationic peptides, exhibited antifungal activity against *Paecilomyces* spp. (10.69 ± 1.11 mm for 0.5 h and 11.29 ± 0.45 mm for 6 h), whereas no inhibition was observed for the P−180 fractions. This observation suggests that cationic peptides may contribute to the antifungal effect and that EDUF may favor their migration toward the P+ compartment. These results are consistent with the known mechanism of action of cationic AMPs, which interact electrostatically with the negatively charged fungal cell membrane, causing membrane disruption and leakage. In contrast, anionic or neutral peptides show limited activity due to weaker binding affinity [43]. The variability observed among fungal strains may be attributed to differences in cell wall composition and charge, as structural components such as chitin, glucans, and mannoproteins influence AMP binding and penetration [44].

Regarding bacterial strains, only the crude hydrolysates at high concentrations of 80 and 40 mg/mL showed inhibition. *L. ivanovii* was the most sensitive, with inhibition zones ranging from 11.50 ± 0.45 mm to 13.17 ± 1.11 mm. Fractions obtained after discoloration and fractionation showed no inhibitory activity in the agar well diffusion tests. Interestingly, in previous studies, discolored hydrolysates exhibited detectable activity in the same assay, whereas in the present study, they did not. The main distinction between the present and previous studies lies in the pH adjustment of the discolored hydrolysates. In earlier work, the pH was maintained at 9, at which the charge state of the peptides differed. Among the 280 identified peptides, more than 200 had a pI lower than 7; therefore, at a pH higher than their pI, such as pH 9, these peptides would carry a stronger negative charge and migrate toward the P− compartment. Consequently, the P+ compartment would be enriched in peptides that retained a positive charge (i.e., those with a pI higher than 9), which could influence their interactions with microbial membranes and, consequently, their antimicrobial activity. In contrast, in this study, the discolored hydrolysates were adjusted to pH 7 to better reflect conditions relevant to meat applications, prevent potential membrane damage, and allow peptide migration according to their charge under near-neutral conditions. This adjustment, however, may have lowered the net positive charge of the peptides, thereby limiting their diffusion and antimicrobial activity. In a study on the effect of pH on the antifungal and antibacterial activities of bovine and porcine hemoglobin hydrolysates, Sanchez-Reinoso et al. reported that alkaline conditions (pH 10) were more effective than neutral (pH 7) or acidic (pH 3) environments [45]. Variations in discolored hydrolysate pH can cause the protonation (at acidic pH) or deprotonation (at basic pH) of amino acid residues within peptides, altering their charge distribution and conformation. These changes can enhance electrostatic interactions between peptides and microbial membranes, thereby promoting their inactivation [46]. Przybylski et al. also investigated the effect of pH (4.7, 6.5, and 9) on the purification and concentration of the AMP α137–141 (TSKYR, 653 Da) derived from bovine hemoglobin using EDUF [36]. They reported that pH 9 yielded the highest purity increase, approximately 75-fold compared with the initial hydrolysate, and enhanced antimicrobial and antioxidative properties in meat preservation. The authors further noted that pH significantly influenced peptide transfer through the ultrafiltration membrane: under acidic conditions, more peptides were positively charged, resulting in higher peptide numbers and concentrations in the recovered fractions, whereas under basic conditions, peptides became more negatively charged, reducing both peptide number and total concentration. Nevertheless, the selectivity of EDUF for peptide α137–141 recovery improved as pH increased due to the charge of the peptide (pI = 10.5). Therefore, maintaining the hydrolysate at pH 9 may be more favorable for enhancing peptide selectivity and preserving antimicrobial activity [36]. The effect of pH on peptide migration was first studied by Poulin et al., who reported that the predominant direction of peptide transport during EDUF depends on the pH of the feed: acidic conditions favor the migration of peptides carrying a net positive charge, while higher pH values increase the migration of peptides that become negatively charged [47].

#### 2.7.2. MIC, MBC and MFC

Table 3 presents the MIC values of the different hydrolysates and fractions against fungal strains. The fractions exhibited the lowest MICs against *R. mucilaginosa*, followed by *Paecilomyces* spp. and *M. racemosus*, with F0-0.5 h and F180-0.5 h showing the strongest activity against all three fungal strains. Moreover, the MFC/MIC ratios (below 4) observed for the fractions tested against *Paecilomyces* spp. and *M. racemosus* indicate a fungicidal effect of these samples. For bacteria (Table 4), the highest activity was observed against *L. ivanovii*, with all hydrolysates and fractions showing activity. In contrast, for *E. coli* and *S. enterica*, activity was detected only in the fractions obtained after EDUF (P+180 or P−180), regardless of the hydrolysis duration. Due to the limited quantity of EDUF fractions available, demineralization was not performed. Since the EDUF fractions contained up to 150 mg/mL of KCl, part of the observed antibacterial and antifungal effects could potentially be attributed to the inhibitory action of this salt. However, in a control test using KCl alone, no inhibition zone was observed in the agar well diffusion assay. In contrast, MICs were observed in the microtitration test, with values of 168 mg/mL against *E. coli*, 81 mg/mL against *L. ivanovii*, 168 mg/mL against *R. mucilaginosa*, and 40.5 mg/mL against *Paecilomyces* spp. The different MIC values obtained for the P+ and P− fractions indicate that the observed inhibition was not solely due to KCl, as the fractions still exhibited weak but measurable intrinsic activity. It is noteworthy that some well-known commercial antimicrobial products used in food applications, such as Nisaplin^®^ or NisinZ^®^, may contain up to 95% salt due to their production process involving salting-out [48,49]. Therefore, the presence of salt does not appear to be a limitation for food applications but rather an advantage, as it may contribute to increasing the overall antimicrobial activity of the product. In addition, the peptides displayed greater activity in liquid media than on agar, which may explain the absence of inhibition zones in the agar diffusion test for certain hydrolysates, such as F0-0.5 h and F180-0.5 h, against *M. racemosus*, despite detectable activity in the microtitration assay. Similar observations have been reported previously, where EDUF treatment of white wastewater protein hydrolysates enhanced antifungal activity against *M. racemosus* in both the anionic and final feed fractions [50]. It has been reported that results obtained from agar diffusion techniques and quantitative MIC methods are not always directly comparable. King et al. [51] showed that hydrophobic compounds may not diffuse efficiently through agar media, which can lead to an underestimation of their antimicrobial activity in diffusion-based assays. Consequently, broth-based MIC methods are generally more appropriate for evaluating the activity of such compounds [51].

The absence or reduction in antibacterial and antifungal activity in the EDUF fractions suggests that the recovered peptides may not possess the structural or physicochemical features required for efficient interaction with microbial membranes. This lack of activity appears to stem less from peptide concentration than from limited affinity for cell surfaces, likely caused by suboptimal charge distribution, inadequate hydrophobicity, or an unfavorable conformation. Even though some peptides carried a net positive charge, their overall charge density and hydrophobic balance may not have been sufficient to drive the electrostatic and hydrophobic interactions necessary to destabilize or disrupt microbial membranes [52]. Furthermore, microbial resistance mechanisms, such as alterations in membrane composition or enzymatic degradation of peptides, may have contributed to the reduced efficacy [53]. Finally, the specific strains tested in this study might be less sensitive than those previously reported in the literature, emphasizing the need to assess additional microbial strains and to optimize peptide structural features to enhance their antimicrobial potential [54].

### 2.8. Potential Bioactivities Inferred from Database Matching

A total of 22 peptide sequences previously reported as bioactive in various databases were identified in different fractions of the chicken hemoglobin hydrolysates (Table 5). These peptides exhibited diverse functionalities, including antioxidative, ACE-inhibitory, antihypertensive, dipeptidyl peptidase (DPP) inhibitory, pancreatic lipase inhibitory, opioid, neuroactive, anticancer, and taste-modulating activities.

Antioxidative peptides such as ALARL, ALI, EAL, LARL, LHA, PHF, PWT, and TPE were among the most frequently identified in the P− and P+ fractions of the 0.5 h and 6 h discolored hydrolysates, respectively. Their low molecular masses (315–542 Da) and GRAVY values (−1.93 to 3.36) are consistent with previously reported antioxidant peptides [55,56] and their migration through the UF membranes. Several studies have also reported the antioxidant potential of chicken and bovine hemoglobin hydrolysates. Zheng et al. identified AEDKKLIQ as a novel antioxidant peptide from chicken red blood cells, and a similar sequence (TAEDKKLIQQA) was abundant in the F0-6 h, F180-6 h, and P−180-6 h fractions of the present study [2]. Likewise, Wang et al. reported HVDPENFKLL as a new antioxidant peptide from bovine hemoglobin hydrolysate [57], while a closely related peptide (HVDPENFRLLGDILIIVLASHF), differing by only one amino acid substitution in a part of that HVDPENFRLL (arginine instead of lysine), was found in the P−180-6 h fraction. Based on these similarities, TAEDKKLIQQA and HVDPENFRLLGDILIIVLASHF could be considered as new potential antioxidant peptides that warrant further investigation.

Several multifunctional peptides, including FDK, IVY, and LSA (mostly abundant in P−180-6 h, P+180-6 h/P−180-6 h/F0 6 h/F180 6 h and P−180-6 h fractions, respectively), exhibited both antioxidative and ACE-inhibitory properties, suggesting their potential contribution to oxidative stability and blood pressure regulation. ACE-inhibitory peptides in hydrolysates of chicken red blood cells were previously demonstrated by Wongngam et al. [58], particularly when alcalase was employed as the hydrolyzing enzyme. In addition, the peptides LGFPTTKTYFPHF and VVYPWT, which possess ACE-inhibitory properties and were obtained from porcine hemoglobin after peptic digestion [3], are closely related to the sequences identified in the present study (VVYPW and VYPWT are mostly abundant in P−180-0.5 h/F180-0.5 h/P−180-6 h and P−180-0.5 h/P+180-6 h/F180-6 h fractions, respectively), differing by only one amino acid residue.

Furthermore, bioactivities related to enzyme inhibition were also observed. Peptides IVYPW, IVYPWT (mostly abundant in P+180-0.5 h and P+180-0.5 h/F180-0.5 h fractions, respectively), and VVYPW were associated with ACE-inhibitor, neuro and DPP III-inhibitory activity, whereas PWTQRF, VLA (mostly abundant in P+180-6 h/F180-6 h and P−180-6 h/F180-6 h fractions, respectively), and VYPWT exhibited DPP IV-inhibitory properties, indicating potential glucose-regulating effects. Recently, YPWTQ, a peptide derived from donkey hemoglobin hydrolyzed by Proteinase K, was also reported to exhibit DPP IV-inhibitory activity [4]. Additionally, VVV and VVYPW (mostly abundant in P+180-0.5 h/P−180-0.5 h and P−180-6 h fractions, respectively) were linked to anticancer and neuroactive functions, respectively. Most identified sequences carried neutral or slightly positive net charges at pH 7, with isoelectric points (pI) ranging from 4.0 to 10.1. Fractions P−6 h, P+6 h, and P+0.5 h exhibited the greatest diversity of known bioactive peptides, highlighting the combined influence of hydrolysis duration and EDUF separation on peptide profile, distribution and final bioactivities. A comparable trend was reported by Cournoyer et al. through electromembrane separation of porcine hemoglobin hydrolysates, where peptide migration was primarily governed by isoelectric point and molecular mass [38]. However, their study, conducted at pH 9, favored the migration of strongly cationic peptides, whereas in the present work (pH 7), both slightly cationic and neutral peptides migrated across the membranes towards the P+ compartment. This difference highlights the strong influence of pH and substrate composition on charge-dependent peptide migration.

**Table 5:** Known bioactive peptides found in the fractions according to database identification. The sequences showed a matching percentage above 85% with the databases.

Sequences	Bioactivity	MW (Da)	GRAVY *	Net Charge at pH 7	pI	Most Abundant Fraction **	Database ***
ALARL	Antioxidative	542.68	1.34	1	9.8	P+180-0.5 h	DFBP
ALI	Antioxidative	315.41	3.36	0	5.5	P−180-6 h	DFBP
ASF	Pancreatic lipase inhibitor	323.34	1.26	0	5.6	P−180-6 h	biopep
EAL	Antioxidative	331.36	0.7	−1	4	P−180-6 h/P−180-0.5 h	DFBP
FDK	Antioxidative, ACE inhibitor	408.45	−1.53	0	5.8	P−180-6 h	DFBP
IVY	ACE inhibitor, Antihypertensive, Multifunctional, and Enzyme inhibitors	393.48	2.46	0	5.5	P+180-6 h/P−180-6 h/F0 6 h/F180 6 h	DFBP
IVYPW	DPP-III-inhibitor	676.81	0.98	0	5.5	P+180-0.5 h	biopep
IVYPWT	DPP-III-inhibitor	777.92	0.7	0	5.52	P+180-0.5 h/F180-0.5 h	biopep
LARL	Antioxidative	471.6	1.22	1	9.7	P+180-0.5 h	DFBP
LHA	Antioxidative	339.39	0.8	0	6.74	P−180-0.5 h	biopep
LSA	ACE inhibitor, Antihypertensive	289.33	1.6	0	5.5	P−180-6 h	DFBP
PHF	Antioxidative	399.45	−0.66	0	7.1	P+180-6 h/F180-6 h	biopep
PWT	Antioxidative	402.45	−1.06	0	5.9	P+180-6 h/F180-6 h	biopep
PWTQRF	DPP-IV inhibitor, Opioid, Multifunctional	833.94	−1.4	1	10.1	P+180-6 h/F180-6 h	DFBP
RLL	Taste	400.52	1.03	1	9.7	P+180-6 h	PlantPepDB
TPE	Antioxidative	345.35	−1.93	−1	4.6	P+180-6 h/P−180-6 h/F0-6 h/F180-6 h	DFBP
VLA	DPP-IV inhibitor	301.38	3.26	0	5.5	P−180-6 h/F180-6 h	DFBP
VVL	ACE inhibitor	329.44	4.06	0	5.45	P+180-0.5 h/P−180-0.5 h	biopep
VVV	Anticancer	315.41	4.2	0	5.5	P−180-6 h	biopep
VVYPW	ACE inhibitor, Neuro, DPP-III-inhibitor	662.78	0.92	0	5.5	P−180-0.5 h/F180-0.5 h/P−180-6 h	DFBP
VYPWT	DPP-IV inhibitor, Opioid, Multifunctional, Antioxidative	664.76	−0.06	0	5.5	P−180-0.5 h/P+180-6 h/F180-6 h	DFBP
YGAET	Stimulating	539.54	−0.82	−1	4	P−180-6 h	DFBP

* Grand average of hydropathy (positive values denote hydrophobic, and negative values denote hydrophilic character). ** These peptides were mostly abundant under the conditions shown in the table, with abundance values derived from the heatmap (Figure 6). *** DFBP [59], biopep [60], and PlantPepDB [61].

## 3. Materials and Methods

### 3.1. Materials

#### 3.1.1. Blood

Blood was collected from chickens immediately after bleeding on an industrial slaughter line (Olymel, Saint-Hyacinthe, QC, Canada). To prevent coagulation, ethylenediaminetetraacetic acid (EDTA; Sigma-Aldrich, Oakville, ON, Canada) was added at 1 g/L of blood while stirring [42]. Pepsin (3200–4000 units/mg protein; P6887) was also obtained from Sigma-Aldrich (Oakville, ON, Canada).

#### 3.1.2. Chemicals

All reagents used for RP-UPLC-MS/MS analyses were of analytical grade and purchased from Sigma-Aldrich (St. Louis, MO, USA). The chemicals used for determining the degree of hydrolysis (DH) included sodium dodecyl sulfate (SDS; Bio-Rad Laboratories Inc., Tokyo, Japan), sodium tetraborate (Fisher Scientific, Ottawa, ON, Canada), o-phthalaldehyde (OPA; Sigma-Aldrich, St. Louis, MO, USA), DL-leucine (Sigma-Aldrich, Oakville, ON, Canada), and β-mercaptoethanol (Sigma-Aldrich, St. Louis, MO, USA). KCl (2 g/L) was obtained from EMD Chemicals Inc. (Port Wentworth, GA, USA), and Na_2_SO_4_ (20 g/L) from ACP Inc. (Montreal, QC, Canada). HCl and NaOH used for adjusting the pH and cleaning the EDUF system were purchased from Fisher Scientific (Montreal, QC, Canada).

#### 3.1.3. Microorganisms and Media

The bacterial strains *Salmonella enterica* ssp. *Newport* ATCC 6962, *Listeria ivanovii* HP B28, and *Escherichia coli* MC 4000 were selected due to their prevalence as poultry meat pathogens and their frequent implication in foodborne illnesses in Canada [62]. Likewise, the fungal strains *Paecilomyces* spp., *Mucor racemosus* LMA-722.5332-9a, and *R. mucilaginosa* 27173 were chosen for their common association with food spoilage. These fungal strains were obtained from the Metabiolac collection at Université Laval [63]. All bacterial and fungal strains were reactivated in Tryptic Soy Broth (TSB; BDBacto™, Franklin Lakes, NJ, USA) and Potato Dextrose Agar (PDA; DIFCO™, Sparks, MD, USA), respectively, for 24 h at 37 °C (bacteria) and 3–5 days at 25 °C (fungi) under aerobic conditions. Other materials used for antimicrobial tests included agar and the antibiotics ampicillin, ciprofloxacin, and natamycin, which were obtained from Criterion (Hardy Diagnostics, Santa Maria, CA, USA) and Sigma-Aldrich (St. Louis, MO, USA), respectively.

### 3.2. Methodology

#### 3.2.1. Chicken Hemoglobin Preparation and Hydrolysis

A 1% (*w*/*v*) hemoglobin protein suspension with a volume of 1800 mL was prepared under continuous magnetic stirring overnight. It was subsequently heated to 37 °C, adjusted to pH 3 using 1 N HCl, and kept for 15 min to ensure complete hemoglobin denaturation (Anachemia, VWR International, Lachine, QC, Canada) [1]. Peptic hydrolysis was carried out for 0.5 h and 6 h using an enzyme-to-substrate ratio of 1:11 mol/mol, while maintaining constant temperature (using a hot-plate stirrer) and pH [34,64]. Based on prior results, the hydrolysis durations were established to provide optimal conditions for scaling up to semi-industrial applications, while maintaining both antimicrobial efficiency and economic viability [1,29,34,42]. During hydrolysis, 5 mL aliquots were collected at the following duration points: 0 min (before enzyme addition), 2.5 min, 10 min, 15 min, 20 min, and 0.5 h from the 0.5 h hydrolysis, and 0 min (before enzyme addition), 2.5 min, 10 min, 15 min, 20 min, 0.5 h, 3 h, and 6 h from the 6 h hydrolysis. In addition, larger samples (150 mL) were taken at the final duration points of both the 0.5 h and 6 h hydrolyses.

After sampling, the enzyme was inactivated by adjusting the pH to 9 with 5 M NaOH and maintaining this condition for at least 30 min [65]. The 5 mL aliquots collected throughout hydrolysis were used to determine the degree of hydrolysis (DH) and for MS/MS analysis (to study enzyme mechanism). The larger final samples (150 mL) were freeze-dried for protein content determination and antimicrobial testing (antifungal and antibacterial assays). The remaining portion was subjected to the discoloration process described in Section 3.2.2.

#### 3.2.2. Discoloration of Hydrolysates

The discoloration step was performed according to the method of Nedjar-Arroume et al. [65]. The purpose of discoloration was to obtain a hydrolysate more suitable for food applications, as the red color from heme groups could alter the physicochemical properties of the final product. Additionally, this step helps reduce the risk of membrane fouling during subsequent hydrolysate fractionation by EDUF [33]. Briefly, immediately after collection during hydrolysis, the pH of the crude hemoglobin was adjusted to 4 with 1 N HCl, and the mixture was stored overnight at 10 °C. It was then centrifuged at 10,000× *g* for 3 min to remove the heme group. The resulting supernatant (discolored hydrolysate) was collected, and after sampling, its pH was readjusted to 7 to prepare it for subsequent fractionation. All samples were freeze-dried and used for further analyses, including protein quantification, MS/MS analysis, fractionation by EDUF, and antimicrobial (antibacterial and antifungal) activity tests.

#### 3.2.3. Fractionation by EDUF Process

Cell configuration

**Figure 7:**
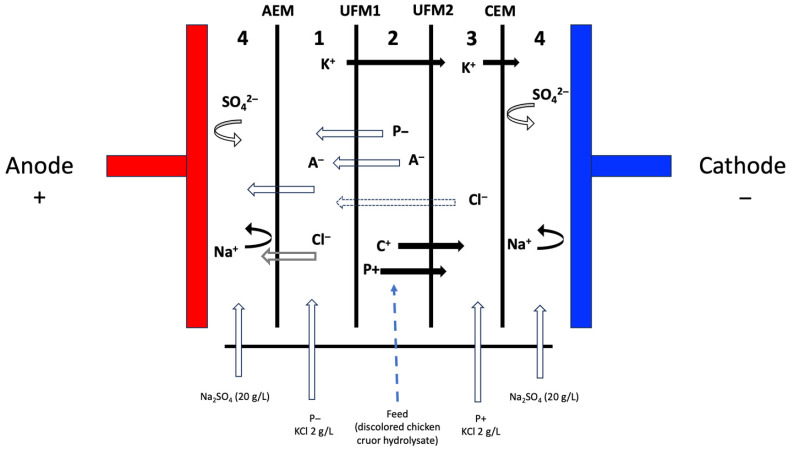
Schematic diagram of the EDUF cell setup. AEM: anion-exchange membrane; UFM: ultrafiltration membrane (50 kDa cut-off); CEM: cation-exchange membrane; P−: anionic peptides; P+: cationic peptides; A^−^: anions.

The electrodialysis system used was a Cell Model MP (Electrocell Systems AB, Stockholm, Sweden) with an effective membrane surface area of 100 cm^2^. The cell was assembled by stacking a food-grade anion-exchange membrane (AEM; Astom, Tokyo, Japan), two polyethersulfone ultrafiltration membranes (UFM1, UFM2; 50 kDa cut-off; Synder, Vacaville, CA, USA), and a food-grade cation-exchange membrane (CEM; Astom, Tokyo, Japan). This configuration was adapted from Kadel et al., specifically incorporating two UF membranes with a 50 kDa molecular weight cut-off (Figure 7) [39].

The distance between the cathode and anode was 3.9 cm, and the cell was divided into five compartments separated by spacers (0.5 cm each) to enhance mixing and turbulence (Figure 7). These five compartments created four independent circulation loops connected to external reservoirs. The first loop (compartments 4) served as a rinsing solution containing Na_2_SO_4_ (20 g/L, 1000 mL), circulated at 800 mL/min. The second and third loops corresponded to the negatively charged recovery compartment after 180 min of EDUF (P−180, compartment 1) and the positively charged recovery compartment (P+180, compartment 3), each containing 650 mL of KCl (2 g/L), circulated at 700 mL/min. The fourth loop (compartment 2) contained 650 mL of discolored chicken hemoglobin hydrolysate (2% protein), also circulated at 700 mL/min. Circulation in all four loops was maintained by centrifugal pumps (Baldor Electric Company, Fort Smith, AR, USA).

##### EDUF Protocol

Before initiating the EDUF runs, the limiting current density (LCD) was determined using the method based on turbulence effects in electrodialysis cells [66]. The applied voltage was then set to 7.8 V (electric field strength of 2 V/cm), a value specifically chosen to ensure that the operating current remained below the LCD during the process [39].

Each EDUF run (three independent replications) was carried out over a three-hour period at room temperature using discolored chicken hemoglobin hydrolysate (0.5 h and 6 h) at a peptide concentration of 2% (*m*/*v*) (Prepared by dispersing the freeze-dried discolored chicken hemoglobin hydrolysate in Milli-Q water and keeping it overnight on a stirrer at 10 °C). Current intensity was directly recorded from the power supply every 15 min. The conductivity of the discolored chicken hemoglobin hydrolysate and of the KCl solutions circulating in the negatively charged (P−180) and positively charged (P+180) recovery compartments was monitored with a YSI conductivity meter (Model 3100; Yellow Springs, OH, USA) and maintained above 3 mS/cm by adding KCl when necessary. The pH of these three compartments was also controlled and maintained at pH 7 using a portable pH meter (Star A221, Fisher Scientific, Ontario, ON, Canada). The pH was chosen to maintain the natural charge of the peptides, as lower or higher pH values (e.g., 3 or 9) would alter their distribution between the P−180 and P+180 fractions [36]. During the process, 2 mL samples were taken from the feed, P+180 and P−180 at 0, 30, 60, 90, 120, 150 and 180 min to quantify peptide concentration and migration rate.

**Figure 8:**
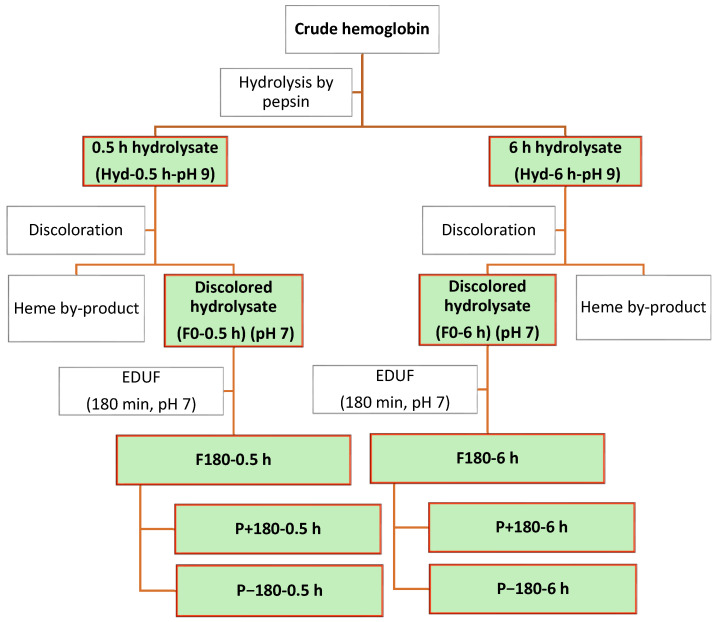
Flowchart of the process and samples subjected to chemical and antimicrobial analyses. F0 (initial feed), F180 (final feed), P+180 (positive-charge peptide recovery compartment), and P−180 (negative-charge peptide recovery compartment). The green boxes indicate the hydrolysates and fractions that were characterized and tested for antimicrobial activity.

The membranes were characterized by measuring thickness and conductivity both before and after the EDUF process. Finally, discolored chicken hemoglobin hydrolysate and its fractions (P+180 and P−180) were further analyzed for peptide sequencing by RP-UPLC-MS/MS and tested for antibacterial and antifungal activities. Figure 8 shows a flowchart of the process and samples subjected to chemical and antimicrobial analyses (in green).

### 3.3. Analyses

#### 3.3.1. Degree of Hydrolysis (DH)

The degree of hydrolysis (DH) was measured using the orthophthaldialdehyde (OPA) assay as described by Church et al. [67], with slight modifications. The OPA reagent was prepared by mixing 100 mM sodium tetraborate, 20% *w*/*w* SDS, 0.160 g OPA, 4 mL methanol, and 400 μL β-mercaptoethanol, then diluting to 200 mL with distilled water. A DL-leucine calibration curve was prepared in 1% (*w*/*v*) SDS. After 2 min of reaction, absorbance was measured at 340 nm, and the degree of hydrolysis (DH%) was calculated as the ratio of free amino groups released during hydrolysis (h) to the total peptide bonds in hemoglobin (8.3 mEq/g protein), accounting for baseline free amino groups (h_0_) and total peptide bonds (h_total_) (Equation 1) [68,69].

(1) $$DH=\frac{h-h_{0}}{h_{total}}\times 100$$

#### 3.3.2. Nitrogen Content

The protein concentration of chicken hemoglobin was determined at four steps: before hydrolysis, after hydrolysis, following the discoloration step, and after EDUF, using the Dumas combustion method with a rapid MicroNcube analyzer (Elementar, Langenselbold, Germany) [70]. A nitrogen-to-protein conversion factor of 6.25 was applied for the calculations [68].

#### 3.3.3. Membrane Characterization

The thickness and conductance of each membrane were measured before and after the EDUF process using an electrical digital micrometer (Marathon Watch Company LTD, Richmond Hill, ON, Canada) and a conductivity meter (Model 3100, YSI, Yellow Springs, OH, USA), respectively. Prior to measurement, all membranes were soaked in 0.5 M NaCl for 30 min to allow equilibration. The electrical conductivity (k) was calculated using Equation (2) [71]:

(2) $$k=\frac{L}{R_{m}\times A}$$

where L is the membrane thickness (cm), A is the electrode area (1 cm^2^), and R*_m_* is the membrane resistance (Ω). The latter was determined according to Equation (3) [71]:

(3) $$R_{m}=\frac{1}{G_{m+s}-G_{s}}$$

where G*_m_*_+_*_s_* is the conductance (S) of the membrane in NaCl solution, and *G_s_* is that of the reference solution.

#### 3.3.4. Peptide Concentration

Peptide concentration in the recovery fraction samples was determined using the Thermo Scientific™ Micro BCA Protein Assay (Waltham, MA, USA) for dilute protein samples. Prior to analysis, all samples were diluted 1/20 with ultrapure water. The working reagent (WR) was prepared by mixing 25 parts of Reagent MA, 24 parts of Reagent MB, and 1 part of Reagent MC (25:24:1, *v*/*v*/*v*). Each diluted sample (150 μL) was mixed with WR (150 μL) in a 96-well microplate, sealed, and incubated at 37 °C for 2 h. After cooling to room temperature, absorbance was measured at 562 nm using a microplate reader (xMark, Bio-Rad, Hercules, CA, USA). Peptide concentration was determined from a bovine serum albumin (BSA) standard curve in the range of 0.5–40 μg/mL, using polynomial regression for curve fitting (R^2^ ≥ 0.99).

#### 3.3.5. Peptides Migration

The total peptide content after 180 min in the recovered solution (g) was used to calculate the migration rate (MR) during the EDUF process according to Equation (4) [24]:

(4) $$MR=\frac{m_{protein}}{S\times T}$$

where MR is expressed in g/m^2^·h, m_protein_ represents the protein mass (g) (from Micro BCA Protein Assay), S is the ultrafiltration membrane surface area (m^2^), and T is the EDUF duration (h).

#### 3.3.6. RP-UPLC and Mass Spectrometry Analyses

Crude and discolored hydrolysates, as well as all fractions obtained after EDUF, were passed through a PVDF filter (0.45 µm; Chromatographic Specialties, Brockville, ON, Canada) and analyzed by reverse-phase UPLC coupled with mass spectrometry, following the protocol of Sanchez-Reinoso et al. [34]. Chromatographic separation was performed on an Agilent 1290 Infinity II UPLC system (Agilent Technologies, Santa Clara, CA, USA), equipped with a binary pump (G7120A), multisampler (G7167B), in-line degasser, and a variable-wavelength detector (VWD G7114B) set at 214 nm. Samples were injected onto a Poroshell 120 EC-C18 column (2.1 × 100 m, 2.7 µm; Agilent, Santa Clara, CA, USA), maintained at 23 °C with a flow rate of 0.5 mL/min and a maximum pressure limit of 600 bar.

The mobile phase consisted of solvent A (LC-MS grade water with 0.1% formic acid) and solvent B (LC-MS grade acetonitrile with 0.1% formic acid). The elution program started at 1% B for 3 min, increased to 13% at 6 min, 35% at 25 min, and reached 100% at 35 min, followed by a 5 min wash at 100% B and re-equilibration to the initial conditions by 47 min.

Peptides were detected and quantified using a 6560 IM-Q-TOF ion mobility quadrupole time-of-flight mass spectrometer (Agilent Technologies, Santa Clara, CA, USA) operated in positive ion mode. Data were collected in Extended Dynamic Range at 2 GHz with a scan range of 100–3200 m/z. Nitrogen served as both the drying gas (13.0 L/min, 150 °C) and nebulizing gas (30 psig). The capillary voltage was set to 3500 V, with nozzle and fragmentor voltages at 300 V and 400 V, respectively. Calibration was carried out with an ESI-L low concentration tuning mix (Agilent Technologies, Santa Clara, CA, USA).

Data acquisition and processing were performed using the Agilent MassHunter software suite (LC-MS Data Acquisition, Version B.09.00, Qualitative Analysis version B.07.00 Service Pack 2 with BioConfirm software). Further processing was performed with MassHunter Profinder (Version B.08.00). Peptides identified in triplicate samples from each hydrolysis duration (0.5 and 6 h), after discoloration (feed initial), and in the fractions obtained after EDUF (feed final, P+, and P−) were matched against an in-house database containing 508 peptides previously characterized from chicken hemoglobin [24].

#### 3.3.7. Antimicrobial Tests

##### Diffusion Assay

The antimicrobial activity of the hydrolysates was evaluated using the agar well diffusion method against selected molds (10^6^ conodia/mL), yeast (10^6^ cells/mL), and bacterial (10^6^ CFU/mL) strains. To prepare the plates, a suspension of microbial cells (250 µL) was mixed with 25 mL of soft agar (1%) maintained at ~50 °C and poured into Petri dishes. Wells were then aseptically punched into the solidified agar, and 80 µL of hydrolysate (40 mg/mL for fungi; 40 and 80 mg/mL for bacteria) was added to each well. Positive controls consisted of Ampicillin (256 µg/mL; MilliporeSigma, Oakville, ON, Canada) for *L. ivanovii* and *E. coli*, Ciprofloxacin (100 µg/mL) for *S. enterica*, and Natamycin (50 µg/mL; MilliporeSigma, Oakville, ON, Canada) for fungal strains [1,72]. Sterile distilled water served as the negative control [1,73]. Plates were incubated at 37 °C overnight for bacterial strains and at 25 °C for 48 h for fungal strains. Hydrolysates showing antimicrobial activity diffused into the agar and inhibited microbial growth; inhibition zone diameters were measured in millimeters [74].

##### Minimum Inhibitory, Bactericidal, and Fungicidal Concentrations

The minimum inhibitory concentration (MIC) of EDUF fractions was evaluated against *E. coli* MC4000, *L. ivanovii* HPB28, and *S. enterica* ssp. Newport ATCC 6962, *Paecilomyces* spp., *M. racemosus* LMA-722.5332-9a, and *R. mucilaginosa* 27173, following established protocols [1,42]. Stock solutions from the feed and recovery fractions were prepared at two concentrations (80 mg/mL and 40 mg/mL protein) for testing against bacterial and fungal strains, respectively. The bacterial inoculum was adjusted to 10^6^ CFU/mL in TSB (Tryptic Soy Broth), and microplates were incubated for 24 h at 37 °C. For molds and yeasts, PDB (Potato Dextrose Broth) was used, with inoculum prepared at 10^6^ conidia/mL for molds and 10^6^ cells/mL for yeasts, according to Vimont et al. [63]. The microplates were incubated for 48 h at 25 °C. Microbial growth was assessed both visually and spectrophotometrically at 595 nm (Infinite^®^ F200 PRO, Tecan Inc., Durham, NC, USA).

#### 3.3.8. In Silico Identification and Characterization of Bioactive Peptides

The most abundant peptides identified under each experimental condition, based on heatmap analysis, were selected for in silico evaluation of their potential antimicrobial properties using the DBAASP database [75]. Additional physicochemical characterization, including molecular mass, net charge, isoelectric point, and hydrophobicity, was performed using complementary peptide databases. Moreover, previously reported bioactive peptides were identified across the different fractions through database comparison [59,60,61].

### 3.4. Statistical Analyses

Statistical analyses were performed using SigmaPlot Version 15.0 (Systat Software, Inc., San Jose, CA, USA). Differences among treatments were evaluated by one-way ANOVA (*p* < 0.05), with Tukey’s post hoc test applied for pairwise comparisons. Also, a *t*-test (*p* < 0.05) was used to compare protein content, membrane conductivity and thickness before and after EDUF, as well as the number of peptides between crude and discolored hydrolysates at each hydrolysis duration. In addition, minimum inhibitory concentration (MIC) and minimum fungicidal concentration (MFC) values were assessed with the Kruskal–Wallis test, followed by Dunn’s multiple comparison test (*p* < 0.05) to determine significant group differences [1,38].

## 4. Conclusions

This study investigated, for the first time, the impact of hydrolysis duration (0.5 h and 6 h) on peptide populations and on the performance of EDUF for the fractionation of such chicken hemoglobin time-related hydrolysates. The results show that EDUF enables peptide separation based on charge, generating fractions with distinct peptide compositions. Discoloration prior to EDUF was necessary to prevent ultrafiltration membrane fouling caused by heme, although it did not significantly influence antifungal activity, which remained more pronounced in the final feed fractions. Regarding antimicrobial activity, antibacterial effects observed in the P+ and P− fractions were weak, regardless of hydrolysis duration, and EDUF did not enhance this activity under the tested conditions. Antifungal activity was detected but was not improved through fractionation, suggesting that the antimicrobial efficacy of the obtained fractions remained limited. Peptide identification revealed 22 previously reported bioactive sequences associated in the literature with diverse functionalities, including antioxidant, ACE-inhibitory, antihypertensive, and enzyme-inhibitory activities. These bioactivities were inferred through database matching and therefore represent candidate functionalities rather than experimentally confirmed activities within this study. The EDUF process generated fractions containing distinct peptide populations, in which peptides associated with different reported bioactivities were detected.

Overall, the findings demonstrate the feasibility of using EDUF to fractionate chicken hemoglobin hydrolysates and highlight this by-product as a source of peptides containing sequences with reported bioactive potential. However, the direct linkage between individual peptides and the measured biological activities remains to be established. Future work should focus on isolating and characterizing specific peptides responsible for the observed activities, validating their biofunctions experimentally, and optimizing process parameters (e.g., pH and membrane configuration) to enhance both peptide selective recovery and targeted bioactivity. In addition, demineralization of EDUF fractions should be performed to better distinguish salt effects from peptide-related activity, followed by re-evaluation of their bioactivity under controlled ionic conditions. Further studies on peptide stability, toxicity, and bioavailability are also required to assess their relevance for food and nutraceutical applications.

## Figures and Tables

**Table 1:** Most abundant potentially antimicrobial peptides (DBAASP database) with some of their characteristics, highlighted in intense red color in the heatmap at different conditions, and representative of a concentration in the fraction.

Peptide Sequence	MW (Da)	Charge at pH7	pI ^†^	GRAVY ^‡^	F0- 0.5 h	F0- 6 h	F180- 0.5 h	F180- 6 h	P+180- 0.5 h	P+180 -6 h	P−180- 0.5 h	P−180- 6 h
AAWQKL	715.9	1	8.80	−0.15					*			
ADKNNVKGIF	1105.3	1	8.63	−0.54						*		
ALARL	542.7	1	9.80	1.34					*			
ALARLL	655.8	1	9.80	1.75					*			
ALTRM	590.7	1	9.80	0.46					*			
AWQKL	644.8	1	8.80	−0.54					*			
DKKLIQQA	943.1	1	8.59	−1.03						*		
DLSHGSAQIKGHGKKVVA	1832.1	2.2	9.70	−0.42							*	
DLSHGSAQIKGHGKKVVAAL	2016.3	2.2	9.70	−0.10							*	
HAHKLRVDPVN	1285.5	1.2	8.76	−0.85		*		*			*	
HALARKY	858	2.1	9.99	−0.79					*			
HGKKVL	680.9	2.1	10.00	−0.57					*			
HKLRVDPVN	1077.3	1.1	8.75	−0.89		*		*		*		
IKNTF	621.7	1	8.75	−0.16						*		
ILGNPMVR	899.1	1	9.75	0.55						*		
ILGNPMVRA	970.2	1	9.75	0.69						*		
KLLG	429.6	1	8.75	0.83						*		
KLLSQ	587.7	1	8.75	−0.12						*		
KQLITG	658.8	1	8.75	−0.03						*		
KQLITGL	772	1	8.75	0.51	*		*		*			
KTYFPHF	939.1	1.1	8.60	−0.73		*		*		*		
LARKYH	786.9	2.1	9.99	−1.22					*			
LARL	471.6	1	9.75	1.23					*			
NFRLLGNIL	1059.3	1	9.75	0.76								*
NIKNTF	735.8	1	8.75	−0.72		*		*		*		
QKVVAGVANAL	1069.3	1	8.75	0.95							*	
RLLG	457.6	1	9.75	0.68		*		*		*		
RVVAHA	651.8	1.1	9.76	0.72					*			
TKTY	511.6	1	8.26	−1.65							*	
TPECQAAWQKLVRVVAHALARKYH	2776.3	3.1	9.78	−0.27								*
VKNL	472.6	1	8.72	0.15		*		*		*		
VKNLDNIKNTF	1305.5	1	8.56	−0.65		*		*		*		
VKNLDNIKNTYAKLSEL	1963.3	1	8.40	−0.59		*		*		*		
VLSAADKNNVKGIF	1475.7	1	8.56	0.26	*		*		*			
VRGHGKKVLGAL	1234.5	3.1	11.17	0.09	*		*				*	
VRVVAHA	750.9	1.1	9.73	1.21					*			
VRVVAHAL	864.1	1.1	9.73	1.54					*			
VRVVAHALA	935.1	1.1	9.73	1.57					*			
VRVVAHALARKYH	1519.8	3.2	11.00	0.09	*		*		*			
WQKL	573.7	1	8.75	−1.13					*			

* Fractions with the highest abundance of specific peptide. † Isoelectric point. **^‡^** Grand average of hydropathy (positive values denote hydrophobic, and negative values denote hydrophilic character). MW: molecular weight.

**Table 2:** Antimicrobial activity of hydrolysates expressed as diameter (mm) of the clear zone around the well containing 40 mg/mL of crude hydrolysates (Hyd), F0 and fraction after EDUF (F180 P+180 and P−180).

	Inhibition Zone Diameter (mm)					
Treatments	*R. mucilaginosa*	*Paecilomyces* spp.	*M. racemosus*	*S. enterica*	*L. ivanovii*	*E. coli*
Hyd-0.5 h	12.15 ± 0.67 ^a^*	13.28 ± 0.47 ^a^	11.13 ± 0.53 ^a^	8.94 ± 0.56 ^a^	12.63 ± 0.84 ^a^	9.42 ± 0.70 ^a^
Hyd-6 h	11.26 ± 0.15 ^a^	14.27 ± 1.31 ^a^	10.22 ± 0.35 ^a^	9.02 ± 0.27 ^a^	11.50 ± 0.45 ^a^	9.72 ± 0.57 ^a^
F0-0.5 h	12.62 ± 0.40 ^a^	14.47 ± 0.19 ^a^	ND	ND	ND	ND
F180-0.5 h	12.59 ± 0.82 ^a^	13.29 ± 0.81 ^a^	ND	ND	ND	ND
F0-6 h	8.34 ± 0.11 ^b^	9.92 ± 0.61 ^b^	ND	ND	ND	ND
F180-6 h	8.66 ± 0.40 ^b^	11.54 ± 0.41 ^b^	ND	ND	ND	ND
P+180-0.5 h	9.41 ± 1.2 ^b^	10.69 ± 1.11 ^b^	ND	ND	ND	ND
P+180-6 h	ND **	11.29 ± 0.45 ^b^	ND	ND	ND	ND
P−180-0.5 h	ND	ND	ND	ND	ND	ND
P−180-6 h	ND	ND	ND	ND	ND	ND

* Means in each column followed by the same letter are not significantly different according to one-way ANOVA followed by Tukey’s post hoc test (*p* > 0.05). ** Not detected.

**Table 3:** The Minimum Inhibitory Concentration (MIC) and Minimum Fungicidal Concentration (MFC) (expressed in mg/mL) of crude hydrolysates (Hyd), F0 and fraction after EDUF (Feed, P+180 and P−180).

	*R. mucilaginosa*			*Paecilomyces* spp.			*M. racemosus*		
Treatments	MIC	MFC	MFC/MIC	MIC	MFC	MFC/MIC	MIC	MFC	MFC/MIC
Hyd-0.5 h	0.04 ± 0.00 ^a^*	0.47 ± 0.22 ^a^	11	0.04 ± 0.00 ^b^	0.31 ± 0.00 ^a^	7	5.00 ± 4.33 ^a^	ND	-
Hyd-6 h	0.16 ± 0.00 ^a^	ND **	-	0.10 ± 0.05 ^ab^	ND	-	13.33 ± 5.77 ^a^	ND	-
F0-0.5 h	0.07 ± 0.02 ^a^	0.63 ± 0.00 ^a^	9	0.10 ± 0.05 ^ab^	0.31 ± 0.00 ^a^	3	1.04 ± 0.36 ^a^	2.08 ± 0.72	2
F180-0.5 h	0.04 ± 0.00 ^a^	1.04 ± 0.36 ^a^	26	0.08 ± 0.00 ^ab^	0.31 ± 0.00 ^a^	3.8	1.25 ± 0.00 ^a^	ND	-
F0-6 h	0.16 ± 0.00 ^a^	ND	-	0.94 ± 0.54 ^ab^	2.50 ± 0.00 ^a^	2.65	>20	ND	-
F180-6 h	0.16 ± 0.00 ^a^	3.75 ± 1.77 ^a^	23	1.25 ± 0.00 ^ab^	1.25 ± 0.00 ^a^	1	>20	ND	-
P+180-0.5 h	0.05 ± 0.02 ^a^	ND	-	10.00 ± 0.00 ^a^	ND	-	20.00 ± 0.00 ^a^	ND	-
P+180-6 h	0.13 ± 0.05 ^a^	ND	-	1.04 ± 0.36 ^ab^	ND	-	15.00 ± 7.07 ^a^	20.00 ± 0.00	1.3
P−180-0.5 h	0.04 ± 0.00 ^a^	ND	-	20.00 ± 0.00 ^a^	ND	-	20.00 ± 0.00 ^a^	ND	-
P−180-6 h	0.04 ± 0.00 ^a^	ND	-	>20	ND	-	20.00 ± 0.00 ^a^	ND	-

* Means with the same letter in each column are not significantly different (Kruskal–Wallis, Dunn’s post hoc; *p* > 0.05). ** Not detected.

**Table 4:** The Minimum Inhibitory Concentration (MIC), Minimum Bactericidal Concentration (MBC) (expressed in mg/mL) of crude hydrolysates (Hyd), F0 and fraction after EDUF (Feed, P+180 and P−180).

	*S. enterica*		*L. ivanovii*		*E. coli*	
Treatments	MIC	MBC	MIC	MBC	MIC	MBC
Hyd-0.5 h	>80	-	15.00 ± 8.66 ^c^	-	>80	-
Hyd-6 h	>80	-	16.67 ± 5.77 ^c^	-	>80	-
F0-0.5 h	>80	-	66.67 ± 23.09 ^ab^	-	>80	-
F180-0.5 h	>80	-	80.00 ± 0.00 ^a^	-	>80	-
F0-6 h	>80	-	53.33 ± 23.09 ^ab^	-	>80	-
F180-6 h	>80	-	40.00 ± 0.00 ^ab^	-	>80	-
P+180-0.5 h	>80	-	33.33 ± 11.55 ^bc^	-	53.33 ± 23.09 ^a^	-
P+180-6 h	80 ± 0.00 ^a^	-	53.33 ± 23.09 ^ab^	-	80.00 ± 0.00 ^a^	-
P−180-0.5 h	>80	-	26.67 ± 11.55 ^bc^	-	66.67 ± 23.09 ^a^	-
P−180-6 h	80.00 ± 0.00 ^a^*	-	20.00 ± 0.00 ^bc^	-	80.00 ± 0.00 ^a^	-

* Means with the same letter in each column are not significantly different (Kruskal–Wallis, Dunn’s post hoc; *p* > 0.05).

## Data Availability

Data will be made available on request to the corresponding author.

## Abbreviations

The following abbreviations are used in this manuscript:

EDUF	Electrodialysis with ultrafiltration membranes
MIC	Minimum inhibitory concentration
MFC	Minimum fungicidal concentration
UFM	Ultrafiltration membrane
Hyd	Hydrolysate
